# Aggressive behavior and metacognitive functions: a longitudinal study on patients with mental disorders

**DOI:** 10.1186/s12991-020-00286-3

**Published:** 2020-06-03

**Authors:** Valentina Candini, Marta Ghisi, Giorgio Bianconi, Viola Bulgari, Antonino Carcione, Cesare Cavalera, Giovanni Conte, Marta Cricelli, Maria Teresa Ferla, Clarissa Ferrari, Laura Iozzino, Ambra Macis, Giuseppe Nicolò, Alberto Stefana, Giovanni de Girolamo, for the VIORMED-2 Group, Stefano Barlati, Assunta Martinazzoli, Giuliana Mina, Roberta Paleari, Francesco Restaino, Bruno Travasso, Antonio Vita

**Affiliations:** 1 Unit of Epidemiological and Evaluation Psychiatry, IRCCS Istituto Centro San Giovanni di Dio, Fatebenefratelli, Via Pilastroni 4, Brescia, Italy; 2 Department of General Psychology, University of Padova, Padova, Italy; 3 Department of Mental Health, ASST Ovest Milanese, Milano, Italy; 4 Training School in Cognitive Psychotherapy, Terzo Centro di Psicoterapia Cognitiva—Scuola Italiana di Cognitivismo Clinico (SICC), Rome, Italy; 5 Department of Psychology, Catholic University of the Sacred Heart, Milano, Italy; 6 Department of Mental Health, ASST Spedali Civili of Brescia, Brescia, Italy; 7 Department of Mental Health, Asst-Rhodense G.Salvini di Garbagnate, Milano, Italy; 8 Unit of Statistics, IRCCS Istituto Centro San Giovanni di Dio, Fatebenefratelli, Brescia, Italy; 9 Department of Clinical and Experimental Sciences, University of Brescia, Brescia, Italy

**Keywords:** Metacognition, Internal mental states, Aggressive behavior, Risk of violence, Mental disorders

## Abstract

**Background:**

Metacognitive functions play a key role in understanding which psychological variables underlying the personality might lead a person with a severe mental disorder to commit violent acts against others. The aims of this study were to: (a) investigate the differences between patients with poor metacognitive functioning (PM group) and patients with good metacognitive functioning (GM group) in relation to a history of violence; (b) investigate the differences between the two groups in relation to aggressive behavior during a 1-year follow-up; and (c) analyze the predictors of aggressive behavior.

**Methods:**

In a prospective cohort study, patients with severe mental disorders with and without a lifetime history of serious violence were assessed with a large set of standardized instruments and were evaluated bi-monthly with MOAS in order to monitor any aggressive behavior. The total sample included 180 patients: 56% outpatients and 44% inpatients, and the majority were male (75%) with a mean age of 44 (± 9.8) years, and half of them had a history of violence. The sample was split into two groups: poor metacognition (PM) group and good metacognition (GM) group, according to MAI evaluation scores.

**Results:**

The PM patients reported a history of violence more frequently than GM patients, during the 1-year follow-up, but no differences between groups in aggressive and violent behavior were found. The strongest predictors of aggressive behavior were: borderline and passive–aggressive personality traits and a history of violence, anger, and hostility. The metacognitive functions alone did not predict aggressive behavior, but metacognitive functions interacted with hostility and angry reactions in predicting aggressive behavior.

**Conclusions:**

This study led to some important conclusions: (a) some aspects closely related to violence are predictive of aggressive behavior only in patients with poor metacognition, thus good metacognition is a protective factor; (b) poor metacognition is associated with a history of violence, which in turn increases the risk of committing aggressive behavior.

## Background

The concept of metacognition refers to an individual’s ability to recognize internal states and, consequently, to build a complete and complex representation of themselves and others, including all elements of human experience, thoughts, emotions, and behavior [15, 57]. The same concept is also used to describe how such representations guide an individual’s actions, especially in difficult situations.

Despite limited and sometimes contradictory data, evidence shows that poor reflective and metacognitive functioning is frequently related to aggression in patients with severe mental disorders (SMD) [1, 10, 30]. It is important to consider that premeditated aggression is associated with relatively intact cognitive, but severely impaired affective metacognitive functions; in contrast, impulsive aggression has been linked to difficulties in both the cognitive and affective processing of mental states (Bo et al. [9]; Bo et al. [7]. Indeed, the relationship between metacognition and violence does not always go in the same direction, but may depend on the type of aggressive behavior (premeditated or impulsive).

In line with the abovementioned study (Abu-Akel et al. [2], Mitchell and colleagues [44] reported lower mastery scores in forensic patients with schizophrenia compared to patients without a history of violence. Their data revealed that both groups performed significantly better understanding their own minds as compared to understanding others’ minds and mastery: even if a small effect size emerged, their data indicated higher scores for understanding others’ minds as compared to mastery. This hierarchical pattern of metacognitive functions is consistent with previous results, whereby it has been proposed that being able to first recognize one’s own mental state will have a strong influence on being able to understand the mental state of others (Lysaker et al. [40].

In the present study, we referred to the explanatory model of metacognition developed by an Italian group of cognitive scientists and psychotherapists (Carcione et al. [16, 48]; Semerari et al. [55, 56]: they posit that metacognition is a complex system, composed of several functions in interaction among themselves, but also partially independent. This approach explains metacognitive functions by distinguishing abilities that represent our own internal states (cognitive, emotional, and motivational) from those regarding the understanding of others’ internal states. The self-domain indicates the way in which a person has explicit access to his/her own mental state (cognitive and emotional) in relation to behavior; it includes monitoring and integrating functions. The other domain refers to skills used to understand the thoughts, emotions, and behavior of others and to differentiate them from their own. This domain includes differentiating and decentering functions (Semerari et al. [56].

This metacognitive model can identify and distinguish skills that may play an important role in triggering violent behavior, such as the difficulty in understanding and expressing one’s own emotions on the one hand, and understanding others’ mental states and their intentions on the other. Indeed, the impairment of these abilities may lead to maladaptive management of interpersonal relationships and to the consequent risk of violence as a conflict-resolution strategy.

## Methods

### Aims and hypotheses

The present study is part of the Violence Risk and Mental Disorders (VIORMED) project (for further details, see [3, 21] submitted). This is a prospective cohort study involving inpatients living in residential facilities (RFs) and outpatients of the Departments of Mental Health in Northern Italy. This specific study aimed to investigate metacognitive functions as potential discriminating factors, underneath other clinical characteristics, between people with SMD who have behaved aggressively and people with the same disorders who have never behaved aggressively. The aims of the study were the following:

1. To investigate the differences between patients with poor metacognitive functioning (PM group) and patients with good metacognitive functioning (GM group) in relation to a history of violence.
2. To investigate the differences between the PM and GM groups in relation to aggressive behavior displayed by patients during a 1-year follow-up (FU).
3. To analyze the predictors of aggressive behavior and to evaluate if metacognitive functions associated with other domains (e.g., personality traits, anger, impulsiveness, hostility, emotional recognition) are related to the emergence of aggressive behavior during a 1-year follow-up.

The main hypothesis of this study is that impaired metacognitive functions lead to an increased risk of violence in patients with SMD. In fact, the ability of these patients to understand their own internal states and those of others is crucial for the effective management of relational problems, and this can also be crucial in avoiding aggressive behavior against other people.

### Participants

Violent patients had to meet one or more of the following criteria: (i) to be admitted at least once to a forensic mental hospital (FMH) for any violent acts against other people; (ii) to be arrested at least once for any violent act against other people; or (iii) to have a documented lifetime history of violent acts against other people (as reported in official clinical records). The control group included patients who did not meet any of these three conditions. Exclusion criteria were being older than 65 years and having a primary diagnosis of an organic mental disorder. This study was approved by the relevant ethics committees, and all participants provided written informed consent.

Of the 290 patients included in the VIORMED study, 98 chose not to participate: indeed, the interview for the assessment of metacognition was audiotaped, and this procedure caused them discomfort, so they declined to participate. We compared the main clinical and sociodemographic features of the refusers and compliers: the only differences had to do with the diagnosis and the patients’ collaboration (assessed with the patients’ schedule). Patients who refused more frequently met diagnostic criteria for schizophrenia compared to patients who accepted (70% vs 47%, *p* = 0.002), and were less cooperative in their treatment (84% vs 93%, *p* = 0.013). There was also a significant gender difference, but it is difficult to draw any conclusion because the sample is highly unbalanced. Among the 192 patients who consented to participate, we had to discard data about 12 subjects because their audiotaped interviews were not suitable for scoring. Therefore, the total sample of this study included 180 patients.

To examine metacognitive functions in relation to the various dimensions investigated, including the primary outcome (aggressive behavior), the patients were split into two groups: patients with poor metacognitive functions (PM group) and patients with good metacognitive functions (GM group). The groupings were based on Likert-scale scores (1 = absent, 2 = poor, 3 = good with help, 4 = very good, 5 = very good and spontaneous) of the Metacognition Assessment Interview (MAI) and on the average scores of our sample (range 1.2–3.6). Also this value is the median and allowed the establishment of two numerically homogeneous groups. As a result, patients with an average total score lower than 2.5 (i.e., the median value) were classified as PM, while those with an average score equal to or greater than 2.5 were classified as GM. In the final sample, made up of 180 patients, 87 patients fell into the PM group and 93 into the GM group.

### Patients with a history of violence

Ninety-six patients (53%) had a lifetime history of violence represented by a documented event of physical aggression against other people. The violent event in 72% of cases was a physical assault, 7% stalking, 4% attempted murder, and 4% murder. The remaining cases involved other types of violent acts against other people. In addition, 35% of the patients had already assaulted other people in the 6 months before the index event. Sixty-five percent of the patients, at the time of the violent act, was already diagnosed with SMD and was in treatment in a MHS. In 30% of the cases, despite the violent act, the victim did not suffer any physical damage; in 50% of the cases, the injuries were moderate; while in 20% of the cases, there were very severe injuries (also leading to death).

In 45% of cases, the victims were family members, in the other 20%, the victims were in close contact with the patient, which included 7% of the cases involving a staff member.

### Clinical assessment

A patient schedule was used to collect information about sociodemographic and clinical characteristics. One section (only for violent patients), concerning their history of violence, was filled out for each patient.

The Structured Clinical Interview for DSM-IV for Axis I (SCID-I) and Axis II (SCID-II), which are semi-structured interviews based on DSM-IV criteria, were used to confirm standardized clinical diagnoses (First et al. [27, 28].

Psychopathology was assessed by the Brief Psychiatric Rating Scale (BPRS) (Ventura et al. [62], which is a rating scale used to measure psychiatric symptoms: each symptom is rated on a scale from 1–7 (the highest scores correspond to more severe symptoms), and a total of 24 symptoms are scored.

Psychosocial functioning was evaluated by the Personal and Social Performance (PSP) scale, a modified version of the DSM-IV Social and Occupational Functioning Assessment Scale (SOFAS) [46]. The PSP scale consists of a single score that ranges from 0 to 100, with higher scores indicating better functioning.

### Metacognition assessment

The Metacognition Assessment Interview (MAI; Semerari et al. [56] is a semi-structured interview that begins with an open question asking the patient to describe a relational negative autobiographical episode (conflicting and/or source of discomfort) that occurred in the last 6 months. To evaluate the patient’s comprehension in relation to another person’s mental state, the episode has to include an interaction with another person. After the patient reported the episode, the interviewer set out predefined questions that sequentially investigated four metacognitive functions: monitoring, differentiating, integrating, and decentering.

Each of these functions is evaluated by four specifiers (related to the questions), to which a score from 1 to 5 is assigned (the higher score indicates more functionality), and the total score of the 4 (sub)functions indicates the general metacognitive functioning and ranges from 16 to 80.

The researchers were specifically trained to conduct the interview. The ICC for Monitoring facets ranges from.54 to 0.69; for Differentiating facets from 0.44 to 0.76; for Integrating facets from 0.59 to 0.64; and for Decentering facets from 0.41 to 0.57. Cronbach’s α for the global scale is 0.91 [56].

### Aggressiveness, impulsivity, and hostility assessment

#### Aggression and impulsivity were evaluated using three self-report instruments

The Buss–Durkee Hostility Inventory (BDHI) is a 75-item true/false questionnaire that assesses eight subscales related to hostility, resentment, and negative affect [13].

The Barratt Impulsiveness Scale (BIS-11) is a 30-item, 4-point Likert scale questionnaire that assesses personality and behavioral impulsiveness; it includes three subscales: cognitive impulsiveness, motor impulsiveness and non-planning impulsiveness [47].

The State–Trait Anger Expression Inventory 2 (STAXI-2) is a self-report questionnaire that includes 11 subscales plus an anger expression index, as an overall measure of total anger expression [58].

### Personality assessment

The Millon Clinical Multiaxial Inventory—III (MCMI-III) is a self-report questionnaire designed to provide a personality profile. It is made up of 175 true–false items [43]. For this study, we focused on the 14 personality disorder scales. These scales assess clinical areas according to DSM-IV diagnostic criteria for PD, with higher scores indicating higher levels of psychopathology.

The MCMI-III uses a base rate (BR) transformation score for raw score conversion [32]. A BR > 84 indicates that the patient endorses all symptoms at the diagnostic level, so a full-blown PD is possible; BR scores from 75 to 84 suggest the presence of clinically significant traits and subthreshold symptoms; BR scores < 75 are generally considered not clinically relevant.

### Emotional recognition assessment

The Facial Expressed Emotion Labeling (FEEL; Kessler et al. [36] test is a reliable and valid performance test for measuring the ability to recognize facially expressed emotions. Pictures of 6 different emotions (anger, fear, sadness, happiness, surprise, and disgust) are presented on a screen for 300 ms.

### Longitudinal monitoring of violent behavior

During the 1-year FU, every 2 weeks the researcher asked the treating clinician or the patient’s caregiver to fill out the Modified Overt Aggression Scale (MOAS; Kay et al. [35] for each patient. The MOAS includes the following 4 subscales of aggression: verbal, against people, against objects, and self-harm behavior. A score from 0 to 4 is assigned to each act, where 0 indicates no aggression and 4 denotes very severe aggression. In each subscale, the score is multiplied by a factor specific for the category, i.e., 1 for verbal aggression, 2 for aggression against objects, 3 for aggression against self, and 4 for aggression against other people. Therefore, the total weighted score ranges from 0 (no aggression) to 40 (maximum grade of aggression); during the 1-year FU with 24 administrations (every 2 weeks) the MOAS total score ranged from 0 to 960. We will subsequently refer to the weighted MOAS score simply as the MOAS score.

### Statistical analyses

Categorical variables (sociodemographic and clinical characteristics, or history of violence) distributions were compared between the two groups, PM patients and GM patients, through the Chi square test (or Fisher exact test if there were cells with a number lower than 5). For quantitative variables (i.e., quantitative sociodemographic and clinical characteristics, BIS-11, BDHI, FEEL, MCMI-III, STAXI-2 and MOAS) normality assumption was verified through visual inspection of boxplots and QQ-plots and by the use of Kolmogorov–Smirnov and Shapiro–Wilk tests. Comparisons were therefore performed through t-tests or Mann–Whitney non-parametric tests, depending on the normality or not of the variables analyzed. These comparisons were checked for potential confounders of sociodemographic and clinical features as well.

Monitoring of violent behavior was performed by analyzing the MOAS total score and MOAS subscales across the 24 time-points during FU through smoothing-splines method [54] for trend estimation.

Predictors of aggressive and violent behavior were tested by performing Generalized Linear Models (GLMs, with Tweedie distribution and log-link function) since the MOAS scores were not normally distributed (skewed and zero-inflated); the MOAS total score was entered as the dependent variable and continuous and categorical measures, as well as interaction between metacognitive group and predictors, included as independent variables.

All tests were two-tailed, with the statistically significance level set at *p* = 0.05. All data were coded and analyzed using the Statistical Package for Social Science (SPSS, version 23) and R: a language and environment for statistical computing (R Core Team [52]).

## Results

### Sociodemographic and clinical characteristics

The total sample of this study included 180 patients, 87 in the PM group and 93 in the GM group: 56% outpatients and 44% living in RFs. Most patients were males (75%), married (62%), with a medium–low educational level (69%), unemployed (71%), and with a mean age of 43.8 years (SD = 9.8).

The two groups did not differ regarding sociodemographic characteristics, except for age, level of education, duration of the disorder, and age at first contact with mental health professionals; patients in the PM group were older and had a lower educational level compared to those in the GM group (Table 1). Furthermore, PM patients showed a longer duration of the disorder and had a later contact with Mental Health Services (MHS) compared to GM patients. Regarding psychopathology and psychosocial functioning (as evaluated, respectively, with the BPRS and the PSP scale, no significant differences emerged between the two groups (Table 2).

**Table 1:** Sociodemographic characteristics of PM and GM patients

	PM (*N* = 87)	GM (*N* = 93)	Test^a^	*p* value
Age				
Mean (SD)	46.67 (10.2)	41.01 (9.36)	*F* = 15.34	< 0.001
Gender				
Male (%)	69 (79.3)	66 (71.0)	*Χ*^2=^ 1.67	0.196
Female (%)	18 (20.7)	27 (29.0)		
Setting				
Outpatients (%)	47 (54.0)	54 (58.1)	*Χ*^2= ^0.3	0.585
Inpatients (%)	40 (46.0)	39 (41.9)		
Marital status				
Single (%)	33 (37.9)	36 (38.7)	*Χ*^2=^ 0.012	0.914
Married or cohabitating (%)	54 (62.1)	57 (61.3)		
Education				
Low (%)	67 (77.0)	57 (61.3)	*Χ*^2=^ 5.18	0.023
Medium–high (%)	20 (23.0)	36 (38.7)		
Employment				
Employed (%)	24 (27.6)	27 (29.7)	*Χ*^2=^ 0.10	0.759
Unemployed (%)	63 (72.4)	64 (70.3)		
Cohabiting				
Alone (%)	26 (33.8)	19 (23.8)	*Χ*^2=^ 5.53	0.063
Family (%)	43 (55.8)	58 (72.5)		
Others (%)	8 (10.4)	3 (3.8)		
Frequent social contacts				
Yes (%)	77 (80.2)	65 (78.3)	*Χ*^2=^ 0.097	0.755
No (%)	19 (19.8)	18 (21.7)		
Time spent doing nothing				
Up to 3 h a day (%)	32 (36.8)	43 (47.3)	*Χ*^2=^ 2	0.157
More than 3 h a day (%)	55 (63.2)	48 (52.7)		
Family support				
Present (%)	58 (69.9)	68 (78.2)	*Χ*^2=^ 1.52	0.218
Absent (%)	25 (30.1)	19 (21.8)		
Treatment collaboration				
Collaborative (%)	78 (90.7)	88 (96.7)	*Χ*^2=^ 2.74	0.098
Not collaborative (%)	8 (9.3)	3 (3.3)		

^a^Chi square test for the categorical variables and ANOVA for quantitative variables

**Table 2:** Clinical characteristics of PM patients and GM patients

	PM (*N* = 87)	GM (*N* = 93)	Test	*p*-value^a^
Disorder duration in years (M, SD)	20.9 (10.09)	16.58 (9.22)	*F *= 5.88	0.016
Age of the first contact with services (M, SD)	30.17 (11.9)	26.78 (8.44)	*F *= 5.18	0.024
Lifetime compulsory admissions				
None (%)	41 (54.7)	46 (56.1)	*F *= 3.44	0.173
1–3 (%)	27 (36.0)	34 (41.5)		
≥ 4 (%)	7 (9.3)	2 (2.4)		
Primary psychiatric diagnosis defined by the clinician				
Schizophrenia (%)	38 (43.7)	41 (44.1)	*Χ*^2 = ^1.58	0.664
Personality disorder (%)	29 (33.3)	26 (28.0)		
Bipolar disorder (%)	9 (10.3)	15 (16.1)		
Anxiety and mood disorders (%)	11 (12.6)	11 (11.8)		
Personality disorders as defined by SCID II				
Cluster A (%)	15 (20.3)	13 (16.5)	*F *= 4.37	0.386
Cluster B (%)	32 (43.2)	29 (36.7)		
Cluster C (%)	2 (2.7)	5 (6.3)		
Other (%)	10 (13.5)	7 (8.9)		
None (%)	15 (20.3)	25 (31.06)		
Lifetime substance abuse				
No (%)	55 (65.5)	46 (53.5)	*Χ*^2 = ^2.53	0.112
Yes (%)	29 (34.5)	40 (46.5)		
Lifetime alcohol abuse				
No (%)	50 (58.1)	56 (62.2)	*Χ*^2= ^0.31	0.580
Yes (%)	36 (41.9)	34 (37.8)		
BPRS				
BPRS_Tot	41.9(16.4)	38.1 (11.8)	*U *= − 1.18	0.237
BPRS_anxiety- depression	8.0 (3.6)	7.9 (3.5)	*U *= − 0.30	0.763
BPRS_hostility- suspicion	6.1 (3.2)	5.2 (2.4)	*U *= − 1.59	0.111
BPRS_ thinking disorders	7.4 (4.7)	6.6 (3.1)	*U *= − 0.53	0.596
BPRS_ withdrawal	6.4 (3.5)	5.8 (2.4)	*U *= − 0.91	0.365
BPRS_ activation	4.3(2.1)	4.0 (1.6)	*U *= − 0.71	0.480
PSP	54.9 (16.6)	57.1 (17.6)	*F *= 0.75	0.387

^a^Chi square test or Fisher’ exact test for the categorical variables and ANOVA for quantitative variables or Mann–Whitney test for continuous non-normal variables

### Metacognition and a history of violence

PM patients reported a history of violence more frequently than GM patients, considering the MAI total score; the total score represents the general functioning of metacognition, consequently this result indicates that patients with a history of violence more frequently showed impaired metacognitive functioning. We then analyzed the differences in specific metacognitive functions, as assessed with the MAI: the sample was again split based on two levels (poor metacognition < 2.5 or good Metacognition ≥ 2.5) for the 4 specific metacognitive functions, and the presence of patients with a history of violence in the two groups was compared for each function. As Table 3 shows, patients with a poor level of metacognition in monitoring, differentiating, and decentering displayed a history of violence more frequently than patients with a good level of metacognition in the same functions.

**Table 3:** Differences in metacognitive functions between patients with and without history of violence

History of violence	PM N (%)	GM N (%)	*Χ*^*2*^	*p*-value
Total metacognition				
Yes	58 (66.7)	38 (40.9)	12.03	0.001
No	29 (33.3)	55 (59.1)		
Monitoring				
Yes	30 (68.2)	66 (48.5)	5.16	0.023
No	14 (31.8)	70 (51.5)		
Differentiating				
Yes	52 (65.0)	44 (44.0)	7.88	0.005
No	28 (35.0)	56 (56.0)		
Integrating				
Yes	50 (60.2)	46 (47.4)	2.95	0.086
No	33 (39.8)	51 (52.6)		
Decentering				
Yes	60 (65.9)	36 (40.4)	11.74	0.001
No	31 (34.1)	53 (59.6)		

### Metacognition and aggressive behavior during 1-year FU

When we compared aggressive behavior during the 1-year FU, no significant differences emerged between the PM and GM groups in mean MOAS scores, for both total scores (PM = 12.5 ± 24.2 vs GM = 15.2 ± 27.6, *U *= 4102.5, *p *= 0.905) and the 4 subscales scores, related to the different types of aggressive and violent behavior: verbal (PM = 5.6 ± 9.8 vs GM = 5.4 ± 8.6, *U *=3837.5, *p *= 0.226), against objects (PM = 2.7 ± 8.0 vs GM = 2.9 ± 5.5, *U *=4094.5, *p *= 0.505), self-aggression (PM = 1.5 ± 6.7 vs GM = 2.2 ± 7.7, *U *=3907, *p *= 0.637), and against people (PM = 2.6 ± 6.6 vs GM = 4.7 ± 13.9, *U *=3986, *p *= 0.418).

Furthermore, to identify the most aggressive patients, we compared participants with a total MOAS > 16 (third quartile) in the two groups (PM and GM), and even in this case, there were no significant differences between the PM and GM patients (PM = 24% vs GM = 27%, *Χ*^*2*^= 0.216, *p *= 0.730).

**Fig. 1:**
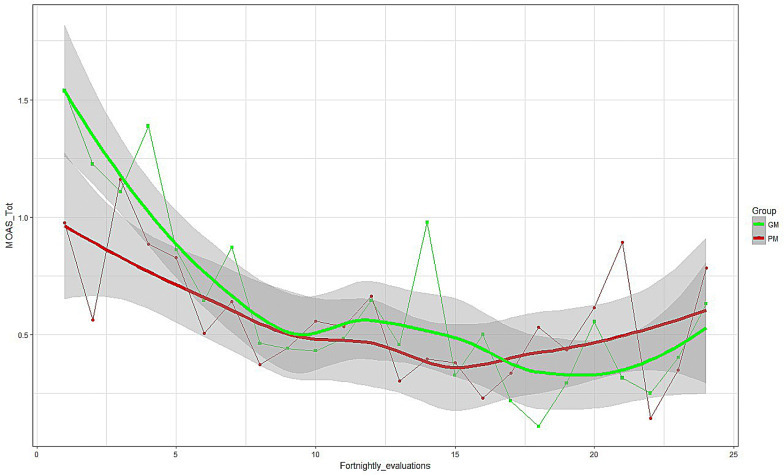
Trends of the MOAS total scores during 1-year follow-up in PM and GM patients

We then analyzed the trends of 24 MOAS ratings (over 12 months) in the two groups, and again we did not find any significant differences. Figure 1 shows MOAS total score trends and their confidence bands (gray bands). The bands of the two groups overlap, and this indicates that the trends of the two groups did not differ (Fig. 1); these trends were both very fluctuating and irregular during the entire FU period.

In addition to the total score, similar analyses were conducted on the four subscales (verbal, aggression against object, self-aggression, aggression against people). The results were the same: there were no differences between the two groups (see Additional file 1: Figs. S1–S4).

### Predictors of aggressive and violent behavior and the role of metacognitive functions

To identify any predictors of aggressive and violent behavior during the 1-year FU, we used GLM on different dimensions: metacognition, a history of violence, care setting, psychopathology, anger, hostility, impulsivity, emotional recognition, and personality traits. In this section, the MOAS score refers to the MOAS total score.

Among all the variables explored (see Table 4), the strongest predictor of aggressive and violent behavior was the presence of borderline and passive–aggressive (negativistic) personality traits; the presence of these traits was associated to a mean increase in MOAS score, respectively, of 211% (exp(β) = 2.11) and 92% (exp(β) = 1.92). At the same time, compulsive and histrionic traits were associated to a mean decrease in the MOAS score, respectively, of 42% (exp(β) = 0.58) and 43% (exp(β) = 0.57).

**Table 4:** Predictive factors of aggressive and violent behavior during 1-year follow-up

	*p*-value	exp(β)
MAI (PM vs GM)	0.376	0.82
History of violence (yes vs no)	0.006	1.86
BDHI		
Total score	< 0.001	1.05
Assault	0.003	1.17
Indirect aggression	< 0.001	1.28
Irritability	< 0.001	1.25
Negativism	0.051	1.17
Resentment	0.001	1.22
Suspicion	< 0.001	1.22
Verbal aggression	< 0.001	1.20
STAXI-2		
Trait anger	< 0.001	1.07
Angry reaction	0.002	1.11
Angry temperament	< 0.001	1.19
Anger expression-out	0.014	1.06
FEEL		
Fear	0.030	0.89
BPRS		
Hostility–suspicion	0.002	1.12
MCMI-III		
Compulsive (yes vs no)	0.018	0.58
Passive–aggressive (yes vs no)	0.008	1.92
Borderline (yes vs no)	0.002	2.11
Histrionic (yes vs no)	0.048	0.57

Similarly, a history of violence was proven to be a very important predictor; the presence of a history of violence led to a mean increase in the MOAS score of 86% (exp(β) = 1.86).

The BDHI total score and all its subscales, except for guilt, also were also predictive factors. With a unit rise of these subscales’ scores, the MOAS score’s mean increases were 5% for the total score (exp(β) = 1.05), 17% for assault (exp(β) = 1.17), 28% for indirect aggression (exp(β) = 1.28), 25% for irritability (exp(β) = 1.25), 17% for negativism (exp(β) = 1.17), 22% for resentment (exp(β) = 1.22), 22% for suspicion (exp(β) = 1.22), and 20% for verbal aggression (exp(β) = 1.20).

The hostility–suspicion subscale of the BPRS was also a predictive factor; the unit rise of this scale produced a mean increase in the MOAS score by 12% (exp(β) = 1.12).

Regarding emotional recognition by facial expressions, the unit increment of fear recognition in the FEEL test was associated to a mean decrease of 11% (exp(β) = 0.89) in the MOAS score.

Finally, several subscales of the STAXI-2 were predictive of changes in aggressive and violent behavior; a unit increase in the following subscales was associated to mean increases in the MOAS scores, specifically 7% for trait anger (exp(β) = 1.07), 11% for angry reaction (exp(β) = 1.11), 19% for angry temperament (exp(β) = 1.19), and 6% for anger expression-out (exp(β) = 1.06).

Metacognition alone did not emerge as a predictive factor of aggressive and violent behavior. Nevertheless, the potential role of metacognitive functions to predict aggressive and violent behavior was evaluated through the analysis of the interaction between these functions (considering both the GM and the PM groups) and all the dimensions assessed in this study.

Metacognitive functions displayed significant interaction with only three variables, notably, BDHI-assault, BDHI-indirect aggression, and STAXI-2-anger reaction (Table 5). Through a more in-depth analysis aimed at clarifying the qualitative and quantitative aspects of this interaction in predicting aggressive and violent behavior, beta coefficients for the two metacognitive groups (PM and GM) were estimated separately (Table 6). Remarkably, the abovementioned variables that interacted with metacognition emerged as significant predictors of aggressive and violent behavior only for patients with poor metacognition. Indeed, in the PM patients the unit increment in BDHI-assault, BDHI-indirect aggression, and STAXI-2-anger reaction scores was associated to a mean increase in the MOAS total score (12.49), respectively, of 36% (exp(β) = 1.36), 53% (exp(β) = 1.53), and 21% (exp(β) = 1.21). Conversely, these variables did not predict aggressive behavior in patients with good metacognition.

**Table 5:** Interaction of metacognitive functions in predicting aggressive and violent behavior

	*p*-value
BDHI assault	0.001*
Metacognition (*groups*)	0.006*
Interaction	0.005*
BDHI indirect aggression	0.000*
Metacognition (*groups*)	0.024*
Interaction	0.017*
STAXI angry reaction	0.003*
Metacognition (*groups*)	0.005*
Interaction	0.012*

* Adjusted values for ‘Age’ and ‘Disorder Duration’ through GLMs

**Table 6:** Variables that interact with metacognitive functions in predicting aggressive and violent behavior in the two groups (PM and GM)

	PM		GM	
	*p* value	exp(β)	*p* value	exp(β)
BDHI Assault	< 0.001	1.36	0.293	1.08
BDHI Inderect aggression	< 0.001	1.53	0.054	1.16
STAXI Angry reaction	< 0.001	1.21	0.287	1.05

## Discussion

### Metacognition and history of violence

The current study demonstrates that patients with poor levels of metacognitive functioning more frequently reported a history of physical violence against other people compared to patients with good metacognitive functioning. Metacognitive impairment might be related to violence in different ways and through different functions (monitoring, differentiating, and decentering).

Patients with poor monitoring functions have difficulties in recognizing, verbalizing, and processing their internal states, especially negative ones, such as those arising from interpersonal conflicts, and have difficulties in referring their emotions to clear thoughts and in relating these mental states with the behavior to act consistently with one’s own goals. For this reason, they might be more likely to display these thoughts and emotions through aggressive physical behavior against others. Acting out their internal states can become the only pathway for patients with poor monitoring to express their feelings and emotions.

Patients with poor differentiating are usually unable to consider alternative points of view to understand the events of daily life. They deem their point of view as the only possible and proper interpretation of reality; moreover, these patients may also confuse their various mental representations with external reality (and this may eventually lead to the emergence of psychotic symptoms), and they may perceive imagined threats as real; therefore, it is evident that these patients may be prone/inclined to violence to defend themselves for real or perceived-as-real reasons.

Finally, patients with poor decentering, who have difficulties in recognizing and comprehending others’ thoughts, emotions, and motivations, are more prone to violent acts, because they always place the focus on themselves and may interpret many situations as hostile and antagonistic to themselves.

These results, indicating a strong association between poor (or absent) metacognitive functioning and a history of physical violence, are consistent with several clinical observations and with previous literature ( [1, 30]. Furthermore, it would be important to consider self-directed violence, such as suicidal behavior. In fact, a good metacognitive functioning might also play a key role in the processing of emotional turmoil that afflicts people attempting suicide, and in the bereavement process of surviving significant others [51].

Although our metacognitive assessment occurred years after the index violent episode, it has demonstrated a robust stability of metacognitive functions [23, 41], and this is also evident in clinical settings. Unless a patient undergoes psychotherapy, which (when treatment is efficacious) may partly improve these skills, metacognitive functions tend to remain stable over time.

The findings of the present study, in agreement with other pieces of research [30, 39, 44], suggest that patients with SMD display an overall impairment in metacognitive functioning. Indeed, in the entire sample, the levels of metacognitive functions were rated within the “poor” (PM group) and “good” (GM group) ranges, but none of the participants received “very good” or “sophisticated” ratings.

### Predictors of aggressive and violent behavior and the role of metacognitive functions

The frequency and severity of aggressive and violent behavior shown during the 1-year FU was not different between the PM and GM patients. These findings were consistent for all four MOAS subscales which point to different types of aggression and violence (verbal, aggression against objects, self-aggression, and aggression against people).

In general, the number and severity of aggressive and violent episodes for all patients was limited; indeed, the MOAS average ratings, over 12 months, was 13.9 (whereas, the theoretical range for 24 ratings goes from 0 to 960) and was, for the most part, represented by reports of verbal aggressive behavior. While this may suggest that patients in treatment at MHSs are not at high risk of aggressive and violent behavior, on the other hand, the overall low number of aggressive and violent episodes may partially explain the lack of significant differences between the PM and GM groups.

A systematic review (Bo et al. [8]) found that metacognition and other dimensions, such as psychotic symptoms, personality factors, and substance use, may indeed be linked to an increased risk of violence. That review suggests that specific metacognitive profiles might be associated with the occurrence of violence in patients with schizophrenia. Moreover, Taubner and colleagues [60] have shown that metacognitive skills are mediators for the risk of violence in adolescents who were victims of child abuse or neglect. The lack of studies aimed at investigating metacognitive functions in patients with other mental disorders in relation to the risk of violence highlights the need for improved efforts in this area.

The strongest predictors of aggressive and violent behavior during the FU were borderline and passive–aggressive personality traits and a history of violence, whereas metacognition alone did not predict aggressive and violent behavior.

The importance of personality traits as risk factors for aggressive and violent behavior has been demonstrated by numerous studies, including our own VIORMED study (Candini et al. [14]). In a sample of patients living in residential facilities, Candini and colleagues [14] found that antisocial personality traits were strong predictors of aggressive behavior. In the same study, in an outpatient sample, several personality traits, including depressive, sadistic, passive–aggressive, schizotypal, borderline, and compulsive traits, were predictors of aggressive and violent behavior (Bottesi et al. [11]).

In a recent meta-analysis, Yu et al. [64] reported that offenders with any PD had two to three times higher odds of being repeat offenders than mentally ill offenders with no PD or offenders with no mental illness. Different studies have shown that that personality disorders most frequently associated with violent behavior are those belonging to cluster B (Bo et al. [9, 45]), particularly antisocial and borderline personality disorders ([34]).

On the contrary, passive–aggressive traits are less associated with the risk of interpersonal violence. Individuals with passive–aggressive traits may be more emotionally unstable and complaining and more often likely to express aggression through indirect behavior followed by expressions of regret (Craig [20]). A possible explanation of our finding, with passive–aggressive traits being a risk factor for aggressive and violent behavior, is that this trait may represent an indirect feature of aggression and is probably less affected by the bias of social desirability, which is typical of self-report measures. The same reason may explain the lack of association between antisocial traits and aggressive behaviors in our study; we may hypothesize that the antisocial traits deliberately hidden in the self-report questionnaire emerged indirectly as the passive–aggressive traits.

Another strong predictor of aggressive behavior is a history of violence. This result is confirmed by the VIORMED project (Bulgari et al. [12]; Candini et al. [14]; de Girolamo et al. [21]) and by several studies on patients with SMD and/or offenders (Fazel et al. [25, 26]; Lund et al. [38]). At the same time, the present study points out that people with a history of violence are characterized more frequently by poor metacognitive functioning compared to patients without such history; this data is supported by other studies [1, 2, 30]. Therefore, although metacognition alone does not seem to predict aggressive behavior, a history of violence, which has been found to predict aggressive behavior, is more frequent in people with metacognitive deficits. Thus, metacognitive deficits are associated with a history of violence, which, in turn, increases the risk of behaving aggressively and violently.

Finally, it should be underlined that a variety of biological factors can be involved in the complex pathways leading to a common, final outcome represented by aggressive and violent behavior. Several studies have abundantly shown the role played by biological factors and offer additional target for preventive and corrective interventions ([50]).

### Hostility, anger, and metacognition

Hostility as a predictor of aggressive and violent behavior is in line with the literature, which shows that the hostility dimension is strictly related to violence (Birkley and Eckhardt 2015 [6, 53]); this result is also consistent with clinical observations. The tendency to perceive the world and individuals as hostile is a feature of psychological functioning that might be a strong predictor of violent behavior (Garofalo et al. [31]). Indeed, if everything is interpreted as a threat, hostility and suspicion are consequent and, in the person’s mind, legitimate, and the attack–defense reaction (even through aggressive behavior) is more likely to occur.

The anger dimension was also found to be a predictor of aggressive and violent behavior. A recent meta-analytic review confirmed a robust relationship between anger and violent behavior [19]; other authors have demonstrated the fundamental role played by anger in the risk of violence [59] and, in this direction, a recent review suggested that anger treatments are moderately effective to reduce aggression [37].

The present study highlights the role of metacognitive functions associated with other variables that predict aggressive behavior. Indeed, metacognitive functions interact with hostility, manifested through direct and indirect aggression, and with angry reactions through aggressive behavior. These two variables emerged as predictors of aggressive behavior only in patients with poor metacognitive functioning, which may mean that these variables are predictive of aggressive and violent behavior only if they are associated with poor metacognition. The latter result is very important because it leads to relevant conclusions, already supported by clinical observations. Some dimensions strongly linked to aggressive behavior, such as hostility and anger, are not predictors per se, but they become risk factors when metacognitive capacities are impaired and the person fails to express and manage such internal states in an adaptive way. This lack of processing and regulation of some internal states through good metacognitive abilities might lead to the emergence of aggressive behavior. At the same time, metacognitive functions might be considered as potential protective factors from the emergence of aggressive behavior, reducing the triggering role of some dimensions (for example, hostility and anger).

In line with these findings, there is also evidence showing that psychosocial and metacognitive skills, such as empathizing and understanding the perspective of others, are associated with reduced aggressive behavior [1, 29, 63].

### Implication for treatment

It seems that metacognitive dysfunctions are related to the risk of violence and hence constitute essential areas to be treated to avoid aggressive behavior. Each patient may have certain deficits and not others because metacognitive functions are correlated, but only partially independent. For this reason, it is important to have precise assessment measurements to identify compromised functions to plan effective personalized interventions to reduce the risk of violence.

The prevention of aggressive behavior, taking metacognitive functions into account, involves two issues. On the one hand, early identification of patients at high risk of aggressive behavior through a precise evaluation of metacognitive dysfunctions, in order to successfully treat them and prevent, whenever possible, violent acts. Deficits in metacognition may also affect help-seeking behavior and extend the Duration of Untreated Psychosis (DUP) [42]. There is a well-known association between prolonged DUP and poorer outcomes [49].

On the other hand, the prevention of reoffending in patients with a history of violence is a fundamental issue. Our study demonstrates that patients with poor metacognitive functioning have more frequently a history of violence than patients with good metacognitive functioning and that in turn, the history of violence is a strong predictor of future aggressive behavior. Thus, and in light of the association between metacognitive deficits and the risk of violence, it appears very appropriate to recommend targeted metacognitive psychological interventions to patients with a history of violence in order to address their metacognitive deficits.

According to a clinical metacognitive approach developed by Carcione et al. [16, 18] and Semerari et al. [55], the therapist can help the patient by means of metacognitive psychological interventions in the following processes: (a) to recognize and elaborate one’s own internal states, both cognitive and emotional, giving personal meaning; (b) to understand what he/she fears and at the same time what he/she wants to achieve in a certain situation; (c) to express one’s own thoughts, emotions, fears, and desires in an adaptive and functional way for his/herself and for his/her society; (d) to integrate all this information into a personal and continuous experience in which the patient recognizes him/herself and, consequently, to implement an adaptive and consistent behavior with this representation; (e) to distinguish between internal reality, constituted by thoughts, images, and dreams, and an external reality, detected through the senses; (f) to consider one’s own point of view as subjective and debatable, not as absolute and universal for everyone; (g) to build another’s point of view, through recognition (or at least the hypothesis) of thoughts and emotions of others and integrate this information into coherent and complex representations concerning others; and (h) finally, to use all the above information to guide behavior toward personal goals and thus to resolve any relational problems in a functional way for the patient and society (peacefully and respectfully).

Certain studies already support the need for treatment addressing metacognitive abilities to improve the psychosocial outcomes of patients with SMD [17, 18, 22, 24, 33]) and patients with SMD and a history of violence [4].

Also Bo and colleagues [10] in their study about patients with schizophrenia and criminal history, suggested that treatment focused on the functional level of metacognition could reduce delusions and strengthen social functioning. Therefore, they underline the importance of intervention designed to enhance patients’ metacognitive abilities, as the more proximal abilities linked to social functioning. Finally, Bateman and colleagues [5], in a recent study on patients with antisocial personality disorder (and comorbidity with borderline disorder), also found that measures of negative mood and general psychiatric symptoms showed significant improvement and better adjustment following the mentalization-based treatment (MBT).

### Limitations

This research presents some limitations. For the longitudinal evaluation of aggressive and violent behavior, the number of patients, and, consequently, the number of aggressive and violent episodes during the FU, was rather limited. Similarly, the length of the FU (1 year) was equally limited; a longer period of observation may lead to higher recidivism among patients with a history of violence and highlight risk factors that were not observable with 1-year FU.

Other limitations are related to the different domains of metacognitive functioning and aggressive behavior, such as child neglect and abuse, psychopathy, and treatment patterns. Alcohol and substance abuse and neuropsychological features have been discussed in other reports.

Nevertheless, this is the first study that has tried to analyze metacognitive function in relation to a prospective observation of aggressive and violent behavior. Although metacognitive functions are difficult to assess, due to difficulties both in eliciting them and in interpreting data, a semi-structured interview, like the MAI, is likely to represent the most appropriate tool to grasp these dimensions because it is sufficiently flexible; on the contrary, self-report measures might be inadequate because they may not be capable of eliciting metacognitive functions in patients with compromised metacognition.

## Conclusions

The violent behavior of patients with SMD is a worldwide public health problem, which demands a substantial amount of staff time and efforts for its management and significantly contributes to an increase in the stigma of mental disorders [61]. For these reasons, it is important to investigate the factors associated with the risk of violence to plan appropriate prevention and treatment. To realize these plans, there is a need to have a detailed understanding of the mechanisms and pathways that lead people with a mental disorder to exhibit aggressive and violent behavior. Only through a careful clarification of these mechanisms and pathways, it will be possible to identify the critical dimensions that need to be targeted and treated to prevent violence.

The literature concerning metacognitive functions and the risk of aggressive and violent behavior is still very limited, consequently, further studies are recommended. The present study wants to add a piece of knowledge in a critical area of clinical psychology and psychiatry.

## Supplementary information

**Additional file 1: Figure S1.** Trends of the MOAS verbal aggression scores during 1-year FU in the PM patients and GM patients. **Figure S2.** Trends of the MOAS aggression against objects scores during 1-year FU in the PM patients and GM patients. **Figure S3.** Trends of the MOAS self-aggression scores during 1-year FU in the PM patients and GM patients. **Figure S4.** Trends of the MOAS aggression against people scores during 1-year FU in the PM patients and GM patients.

## Data Availability

The datasets used and/or analyzed during the current study are available from the corresponding author on reasonable request.

## Abbreviations

PM: Poor metacognition group
GM: Good metacognition group
