# Supplementary material for: Aggressive behavior and metacognitive functions: a longitudinal study on patients with mental disorders
Source: Ann Gen Psychiatry. 2020 Jun 3;19:36. doi: 10.1186/s12991-020-00286-3 (PMC7271462; doi:10.1186/s12991-020-00286-3)
Supplement: Supplementary file 1 — Additional file 1: Figure S1. Trends of the MOAS verbal aggression scores during 1-year FU in the PM patients and GM patients. Figure S2. Trends of the MOAS aggression against objects scores during 1-year FU in the PM patients and GM patients. Figure S3. Trends of the MOAS self-aggression scores during 1-year FU in the PM patients and GM patients. Figure S4. Trends of the MOAS aggression against people scores during 1-year FU in the PM patients and GM patients. [file 12991_2020_286_MOESM1_ESM.docx]

**Additional file 1**

**Figure 1S. Trends of the MOAS verbal aggression scores during 1-year FU in the PM patients and GM patients**

**
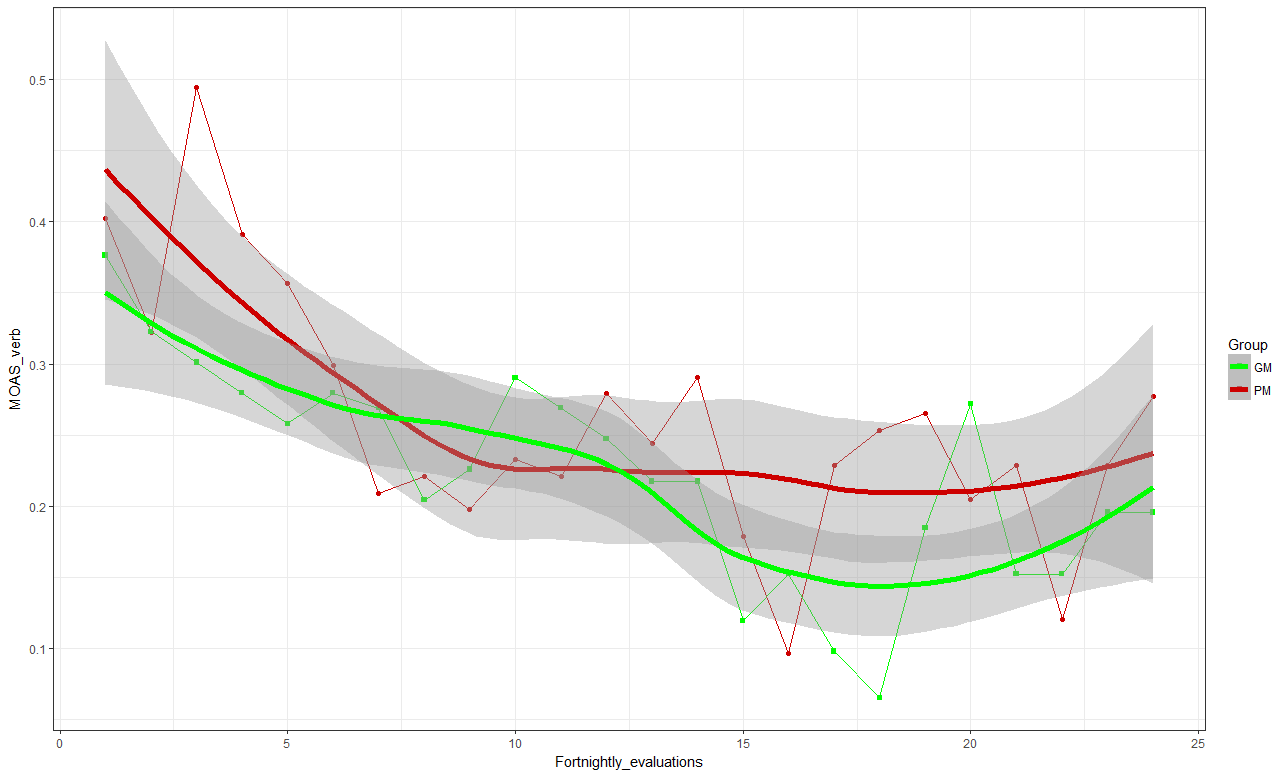
**

**Figure 2S. Trends of the MOAS aggression against objects scores during 1-year FU in the PM patients and GM patients**

**
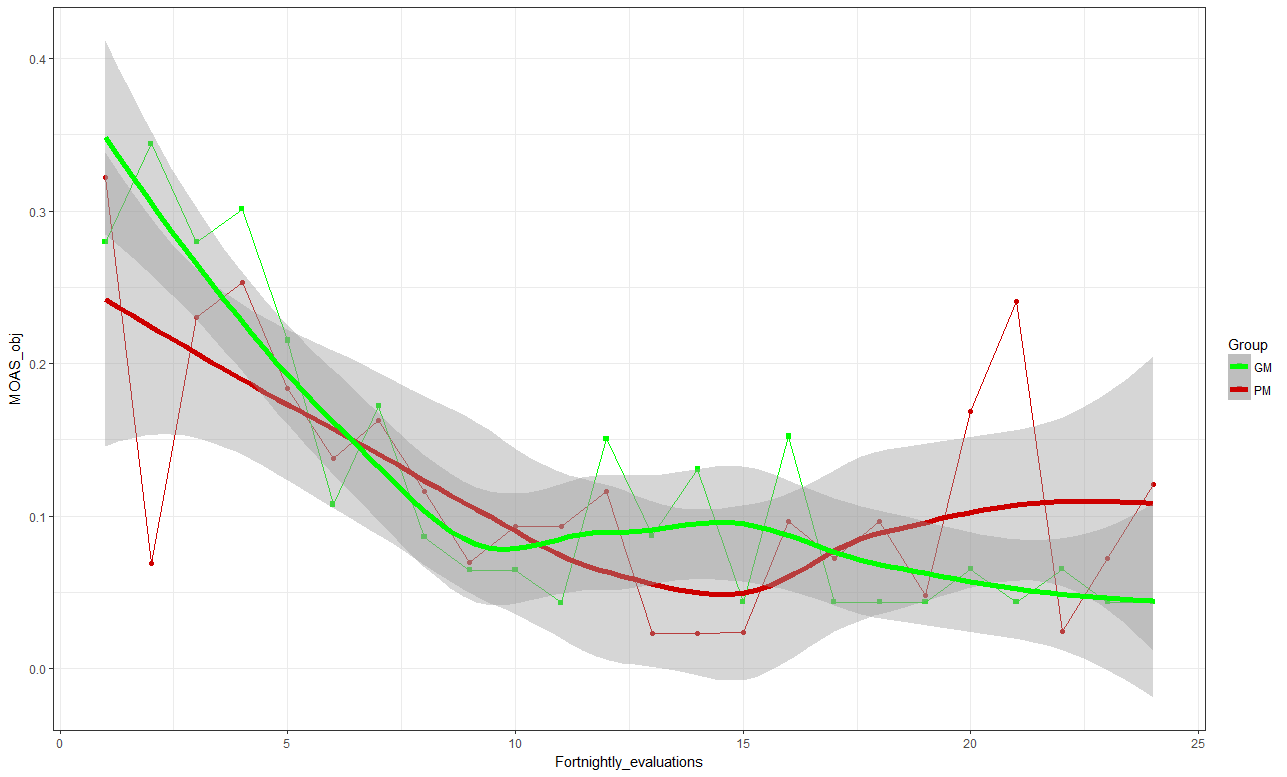
**

**Figure 3S. Trends of the MOAS self-aggression scores during 1-year FU in the PM patients and GM patients**

**
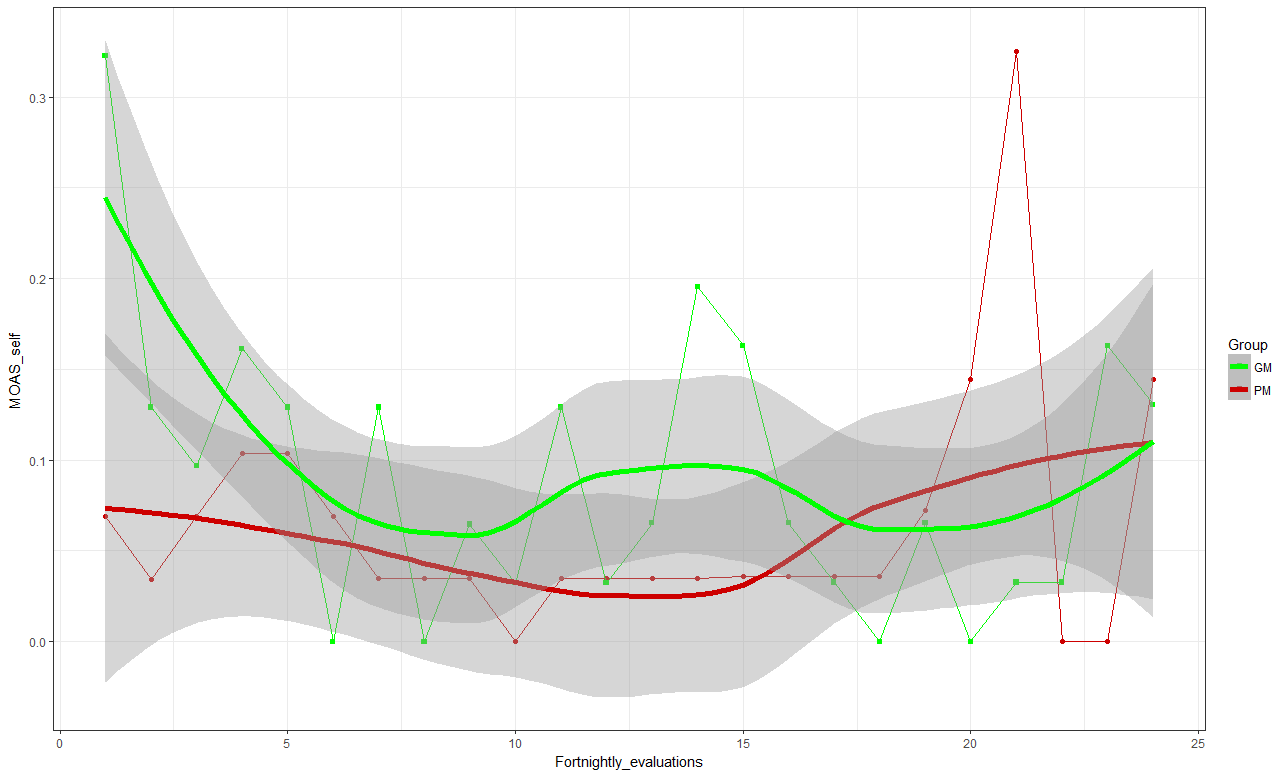
**

**Figure 4S. Trends of the MOAS aggression against people scores during 1-year FU in the PM patients and GM patients**

**
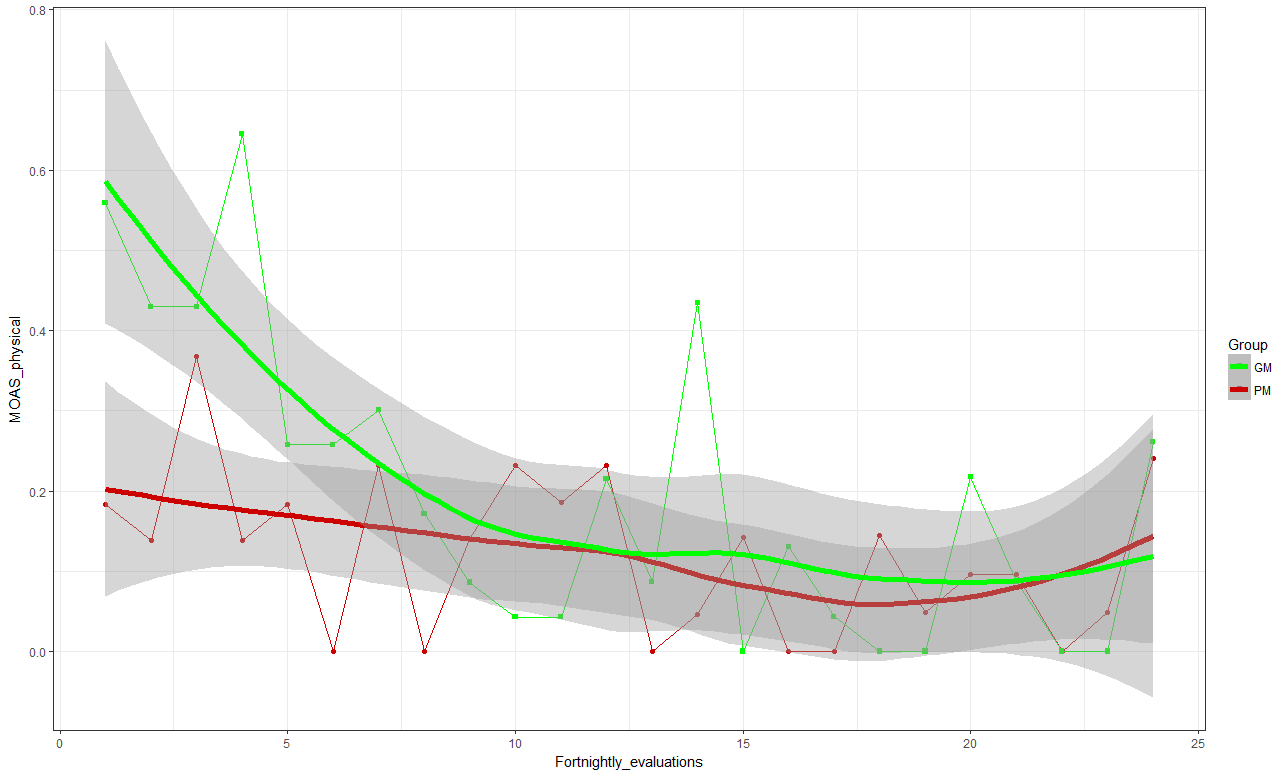
**
